# Li-Nafion Membrane Plasticised with Ethylene Carbonate/Sulfolane: Influence of Mixing Temperature on the Physicochemical Properties

**DOI:** 10.3390/polym13071150

**Published:** 2021-04-03

**Authors:** Aigul S. Istomina, Tatyana V. Yaroslavtseva, Olga G. Reznitskikh, Ruslan R. Kayumov, Lyubov V. Shmygleva, Evgeny A. Sanginov, Yury A. Dobrovolsky, Olga V. Bushkova

**Affiliations:** 1 The Institute of Solid-State Chemistry, Ural Branch of the Russian Academy of Sciences, 620990 Ekaterinburg, Russia; istomina@ihim.uran.ru (A.S.I.); yaroslavtseva@ihim.uran.ru (T.V.Y.); olgarezn@ihim.uran.ru (O.G.R.); 2 The Institute of Problems of Chemical Physics, Russian Academy of Sciences, 142432 Chernogolovka, Russia; kayumov@icp.ac.ru (R.R.K.); shmygleva@icp.ac.ru (L.V.S.); sanginov@icp.ac.ru (E.A.S.); dobr@icp.ac.ru (Y.A.D.)

**Keywords:** lithiated Nafion, ethylene carbonate, sulfolane, swelling, X-ray diffraction, differential scanning calorimetry

## Abstract

The use of dipolar aprotic solvents to swell lithiated Nafion ionomer membranes simultaneously serving as electrolyte and separator is of great interest for lithium battery applications. This work attempts to gain an insight into the physicochemical nature of a Li-Nafion ionomer material whose phase-separated nanostructure has been enhanced with a binary plasticiser comprising non-volatile high-boiling ethylene carbonate (EC) and sulfolane (SL). Gravimetric studies evaluating the influence both of mixing temperature (25 to 80 °C) and plasticiser composition (EC/SL ratio) on the solvent uptake of Li-Nafion revealed a hysteresis between heating and cooling modes. Differential scanning calorimetry (DSC) and wide-angle X-ray diffraction (WAXD) revealed that the saturation of a Nafion membrane with such a plasticiser led to a re-organisation of its amorphous structure, with crystalline regions remaining practically unchanged. Regardless of mixing temperature, the preservation of crystallites upon swelling is critical due to ionomer crosslinking provided by crystalline regions, which ensures membrane integrity even at very high solvent uptake (≈200% at a mixing temperature of 80 °C). The physicochemical properties of a swollen membrane have much in common with those of a chemically crosslinked polymer gel. The conductivity of ≈10^−4^ S cm^−1^ demonstrated by Li-Nafion membranes saturated with EC/SL at room temperature is promising for various practical applications.

## 1. Introduction

Along with its analogues (such as Flemion (Asahi Glass), Fumapem (Dow Chemical, 3M, FuMA-Tech), Aquivion (Solvay), etc.), the perfluorinated sulfonic-acid (PFSA) ionomer Nafion^®^ developed by DuPont in the 1960s is widely used in polymer electrolyte membrane fuel cells, sensors, vanadium flow batteries, ionistors, as well as various other electrochemical devices and electrochemical technologies [1,2,3]. The widespread applications of this ionomer material are due to its excellent transport properties, high thermal and chemical stability, combined with stable mechanical behaviour. Ion exchange reactions can be used to convert PFSA ionomers from their original acid form to a salt form using various cations without loss of strength or thermal and chemical stability [4,5,6,7,8,9,10,11,12,13,14]. These properties allow PFSA salts to be used as polymer electrolytes with unipolar cation conductivity. Single-ion cationic transfer eliminates the concentration gradient in an electrochemical cell along with its problematic consequences [15,16]. At present, the lithium form of the membrane is of particular interest for simultaneously application as electrolyte and separator in lithium-ion [17,18,19,20,21] and lithium-sulfur [22,23,24] batteries.

**Figure 1:**
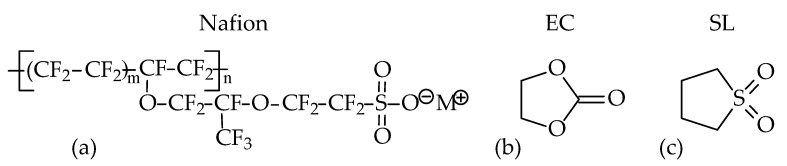
Structural formulas of Li-Nafion (where M^+^ is an exchangeable counterion) (**a**), ethylene carbonate (**b**), and sulfolane (**c**).

Nafion is a random copolymer of tetrafluoroethylene, comprising a semicrystalline polytetrafluoroethylene (PTFE) backbone and a perfluorovinyl ether-bearing terminal sulfonic group (Figure 1a). The outstanding properties of these materials are due to the dissimilar nature of the covalently bounded pendant ionic group (SO_3_^−^) and perfluorinated backbone, resulting in a phase-separated nanostructure [1]. Ionic species in Nafion aggregate to form hydrophilic ionic domains (clusters) distributed evenly within a hydrophobic perfluorinated polymer matrix comprising both amorphous and crystalline regions. Similar to the initial protonated form, the lithiated Nafion (Li-Nafion) membrane is poorly conductive in its dry state. However, upon saturation with dipolar aprotic solvents, a polymer electrolyte offering good transport properties can be obtained [4,5,6,7,8,9,10,11,12,13,14,17,18,19,20,21,22,23,24]. The introduction of solvent molecules enhances nanoscale phase separation. The morphology of swollen Nafion is typically described by the Gierke cluster-network model [25] or equivalent approaches as summarised in a comprehensive review of Kusoglu [1]. As emphasised in Ref. [1], “the sorption behaviour of PFSA ionomers is a very important phenomenon affecting their phase-separated morphology and, hence, their overall transport and structural properties”.

However, lithium-conducting membrane requirements for lithium batteries are not limited to high conductivity, mechanical strength, and chemical stability. For the practical usage of new polymer electrolytes, especially in high-power lithium-ion batteries (LIBs), equally important factors are a wide range of operating temperatures (to at least include ambient temperatures), resistance to local overheating, absence of gassing and electrochemical stability. These factors allow the safety of an LIB to be significantly increased, justifying the replacement of liquid electrolyte onto a polyelectrolyte membrane. However, the development of a suitable plasticiser remains an urgent problem, preventing the full potential of Li-Nafion membranes from being realised.

In recent work [26], we showed that suitable plasticisers for lithiated Nafion can be obtained among binary mixtures of solvents combining high boiling points and flashpoints with low vapour pressures and relatively high melting points (above room temperature). In particular, binary mixtures of ethylene carbonate (EC) and sulfolane (SL) were used to plasticise a lithiated Nafion 115 membrane. In the best sample, single-ion lithium conductivity of 6.3 × 10^−6^ to 6.9 × 10^−4^ S cm^−1^ was achieved within a temperature range of −40 to +80 °C, with a negligible electronic conductivity (≈10^−9^ S cm^−1^ at 25 °C) contribution and an electrochemical stability window of 0 to 6 V vs Li/Li^+^. For a single-ion Li^+^ conductor, σ ≥ 10^−5^ S cm^−1^ is acceptable for practical use in electrochemical cells [15,16]. Thus, a Li-Nafion membrane swollen with the EC/SL mixture seems suitable for practical applications in LIBs.

In the present study, we evaluated the influence of mixing temperature (40 to 80 °C) on the swelling degree of Li-Nafion in binary plasticiser EC/SL and revealed a hysteresis between heating and cooling modes. Following its saturation with a plasticiser, while the crystalline regions remained nearly unchanged, a re-organisation of the membrane’s amorphous structure was observed. These results provide an insight into the physicochemical nature of Li-Nafion membranes swollen with aprotic solvents.

## 2. Materials and Methods

### 2.1. Membrane Preparation

**Figure 2:**
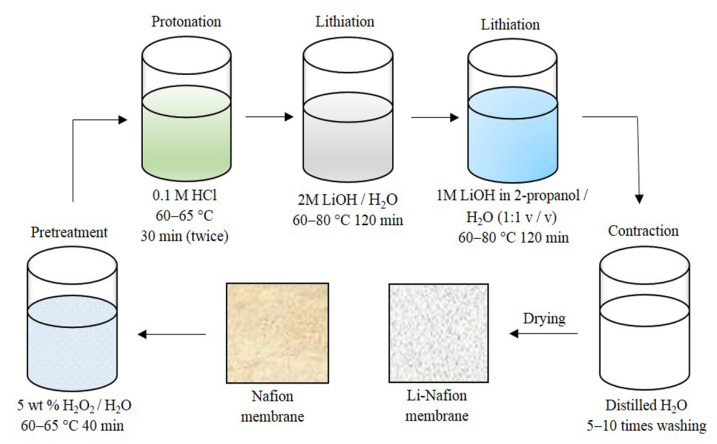
Scheme of the Nafion membrane lithiation process.

In this work, a commercial Nafion^®^ 115 (125 µm) (Du Pont de Nemours, Wilmington, DE, USA) extrusion membrane was used. The Li^+^ form of the membrane was prepared as described in our previous work [9] and shown in Figure 2 over the course of four consecutive stages: purification, neutralisation by LiOH, thorough washing and drying. The samples were first dried in air at 60 °C for 1 h and then in a desiccator at room temperature over P_2_O_5_ for seven days. Residual water content was monitored by infrared Fourier spectroscopy (Bruker Vertex 70v, Bruker Corporation, Karlsruhe, Germany) and simultaneous thermal analysis (STA 449 F3 Jupiter, NETZSCH, Selb, Germany).

All further manipulations of the Li-Nafion membrane and plasticisers were performed in an MBRAUN UNILAB glovebox (MBraun Inertgas-Systeme, München, Germany) in an argon atmosphere (the content of H_2_O and O_2_ < 1 ppm).

### 2.2. Binary Plasticiser Preparation

A binary EC/SL plasticiser was prepared from anhydrous ethylene carbonate (99%) and sulfolane (99%), both from Sigma Aldrich (St. Louis, MO, USA). The mass fraction of sulfolane (ω(SL)) in the EC/SL mixtures varied from 0 to 1 with an increment of 0.05–0.10. Since the melting points of ethylene carbonate and sulfolane are above room temperature (≈36 and ≈29 °C, respectively), they were heated until wholly melted (up to 40 °C for EC and up to 30 °C for SL). Then, the mixtures were stirred for 30 min using a magnetic stirrer and kept at room temperature for at least 24 h before use.

### 2.3. Membrane Swelling

The swelling of the Li-Nafion membrane was investigated gravimetrically; each experiment was repeated in triplicate. A dry membrane sample was placed in an excess volume of solvent (EC, SL, or EC/SL) over activated 3Å molecular sieves. To achieve an equilibrium degree of swelling, the samples were held under isothermal conditions for 24 h (from 40 to 80 °C). The initial and final sample weights were measured. To remove the surface solvent, the samples were blotted dry before measurements. The weight uptake *W* (%) was calculated as:

*W* = (*m*_sw_ − *m*_dry_)/*m*_dry_ × 100%, (1)

where *m*_dry_ and *m*_sw_ are weights of membrane samples before and after swelling, respectively. The uncertainty of *W* measurement did not exceed 1–1.5%.

The molar uptake of solvent λ (equal to the number of solvents molecules per lithium cation) was calculated from *W* values using the formula:

λ = (*W* × EW)/(*Mr*_solv_ × 100%), (2)

where EW is the equivalent weight of the dried Li-Nafion samples (equal to 1106) and *Mr*_solv_ is the average molar mass of the used binary solvent.

### 2.4. Thermal Analysis

The thermal behaviour of the samples was investigated using a DSC 214 Polyma differential scanning calorimeter (NETZSCH, Selb, Germany). A test sample was cut out of the membrane in the form of a disk, placed into a special Concavus Al pan, and hermetically sealed. The measurements were performed under dry nitrogen flow at 40 mL/min within a temperature range from −150 to 300 °C. The following temperature programme was used: heating to 50 °C and isothermal exposure (15 min) for additional homogenisation of the sample; cooling to −150 °C at 20 °C/min and isothermal exposure for 3 min; heating to 300 °C at 40 °C/min.

### 2.5. Wide-Angle X-ray Diffraction (WAXD)

Wide-angle X-ray diffraction data were collected using a Stoe STADI-P diffractometer (Stoe, Darmstadt, Germany) at following conditions: Cu K_α_ radiation; 2θ = 5–90°; rotating sample; symmetrical transmission mode. In order to prevent water contamination, the membrane sample under study was placed between two lavsan films.

A baseline correction was applied to the data prior to further analysis. The measured profile was decomposed into the fundamental profiles by a least-squares fit, with Lorentz functions used to approximate each fundamental profile. The position, height, width, and area of obtained peaks were evaluated.

The degree of crystallinity *X*_c_ (%) was determined for “dry” membrane samples from XRD data according to the following formula:

*X*_c_ = *A*_c_/(*A*_a_ + *A*_c_) × 100%, (3)

where *A*_c_ is the integrated area of the Bragg peak (100); *A*_a_ is the integrated area of the amorphous halo superimposed on the crystalline peak.

### 2.6. AC Conductivity Measurements

Through-plane lithium conductivity was measured at a frequency range 1 Hz to 100 kHz using a Z-350M analyser (Elins LLC, Chernogolovka, Russia) with a potential amplitude of 10 mV. The membrane samples (10 mm in diameter) were placed between two blocking Ni electrodes within a hermetically sealed two-probe test cell and equilibrated for 24 h at 25 °C before measurement. The electrolyte resistance *R_e_* was determined by intercepting the high-frequency part of the impedance curve with the real axis. The specific conductivity σ of the samples was obtained as follows:

σ = *d*/(*R*_e_ × *A*), (4)

where *d* and *R*_e_ are the thickness and resistance of the sample, respectively; while *A* is the area of electrical contact. Conductivity was determined by impedance spectroscopy with an error about 10%.

## 3. Results and Discussion

### 3.1. Characterisation of the Plasticiser

An important point in selecting EC and SL as components of the binary plasticiser was the obvious similarity of their molecular structure (Figure 1b,c). Both of these solvents readily dissolve lithium salts due to the intermediate (SL) and high (EC) dielectric constants (60 and 90, respectively [27]), high dipole moment (4.93 for EC and 4.81 for SL [28]), and the presence of electron donor groups capable of coordinating the lithium cation (Figure 1b,c). This explains the effective solvation of Li^+^ in lithiated Nafion upon swelling with EC, SL, or an EC/SL mixture. The combination of high boiling points with low vapour pressure at *T* < 100 °C (0.003 and 0.009 kPa, respectively [29,30]) and high flash points (145.5 ± 4 and 165 ± 4 °C [30]) offered by both EC and SL solvents is an important factor in improving the safety of LIBs.

While EC and SL solvents have individual melting points above room temperature (36.4 and 28.5 °C, respectively [28]), the mixture formed therefrom offers a significant decrease in the melting point of the plasticiser. Indeed, as established in our recent work [31], the EC–SL system belongs to a simple eutectic type with a eutectic temperature of—16 °C and a eutectic point at 70 wt % SL. Moreover, binary EC/SL mixtures in a moderate range of compositions 50 to 75 wt % SL are prone to transition into the metastable state of a supercooled liquid without crystallisation of components (i.e., becoming glassy) [31], which makes them nearly ideal plasticisers. As shown in our previous work [26], it is advisable to saturate membranes with ES/SL binary plasticiser at temperatures ≥40 °C when both EC and SL components are in a liquid state.

### 3.2. Influence of Plasticiser Composition on Solvent Uptake and Conductivity

**Figure 3:**
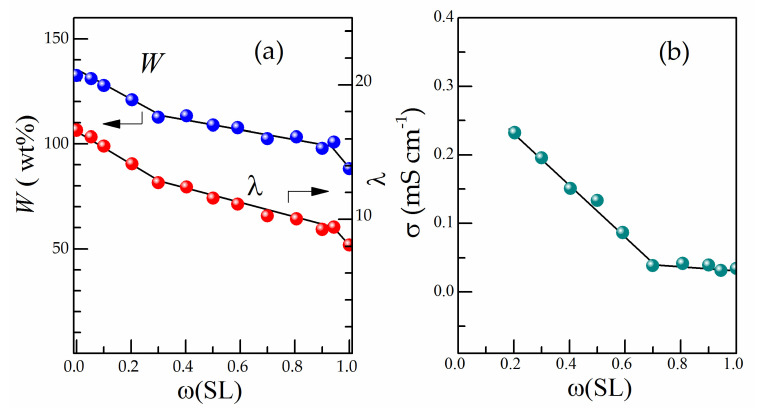
Weight uptake (*W*) and molar uptake (λ) (**a**) and conductivity of swollen Li-Nafion membrane at 25 °C (**b**) as a function of ω(SL) (mixing temperature of 40 °C).

The membranes were saturated with EC/SL binary mixtures of varying composition at 40 °C. The solvent uptake was defined by the gravimetric technique as described in Section 2.3. Figure 3a illustrates the dependence of the weight uptake *W* (calculated by Equation (1)) vs. ω(SL). It can be seen that the highest *W* value relates to the pure EC, while the solvent uptake decreases as ω(SL) increases from 132.5% (pure EC) down to 88.1% (pure SL).

On the assumption that the mixed solvent composition absorbed by Li-Nafion is identical to the initial mixture used (no selective sorption), the total number of binary plasticiser molecules per Li cation in the swollen Li-Nafion membrane (molar uptake λ) was calculated using Equation (2). As can be seen from Figure 3a, the *W* and λ values decrease rapidly as ω(SL) increases, while the λ values differ twice for pure solvents EC and SL (λ = 16.6 and 8.1, respectively). Here, it is important to note that the λ values are much higher than the usual solvation number of free lithium cation equal to 4. The solvation of anions in dipolar aprotic solvents is known to be much weaker than cations. From the above, it follows that the sorption of solvent occurs not only due to the solvation of lithium cations by the highly polar EC and/or SL molecules but also due to some other changes in the membrane.

Figure 3b shows the conductivity isotherm at room temperature for Li-Nafion membranes saturated with EC/SL mixtures of varying compositions at a mixing temperature of 40 °C. For samples saturated with the EC/SL mixtures enriched with ethylene carbonate (ω(SL) < 0.2), it was not possible to measure the conductivity: on cooling from 40 to 25 °C, an excess amount of EC released from the membrane (syneresis) was solidified on the membrane surface. This phase separation agrees with the phase diagram of the EC–SL system [31]: liquidus temperatures for EC/SL mixtures with ω(SL) < 0.2 are above 25 °C. Thus, the non-conductive layer of solid plasticiser forming between the membrane and electrodes makes impedance measurements impossible. The best transport properties of Li-Nafion provided EC/SL mixtures enriched with ethylene carbonate. The membrane conductivity rapidly decreases with ω(SL) in the range of 0.2 to 0.7 (eutectic composition), but then it remains practically unchanged up to pure sulfolane (Figure 3b). Although the decrease in conductivity may be caused by an increase in viscosity with SL content in the mixture [32], this can also be explained in terms of changes in ionic equilibria with the EC/SL mixture composition (since EC and SL differ in solvating ability [26]) or a decrease in the solvent uptake (Figure 3a) influencing the conduction channels within the membrane [1].

### 3.3. Influence of Mixing Temperature on Solvent Uptake

In order to study the influence of the mixing temperature on the solvent uptake of Li-Nafion, pure EC and SL were selected along with binary EC/SL mixtures of two compositions: ω(SL) = 0.2 and 0.7. The choice of these binary compositions was due to the EC-enriched mixture with ω(SL) = 0.2 providing the highest conductivity values of Li-Nafion (Figure 3b) and being homogeneous at room temperature, which prevents phase separation upon cooling, while the mixture with ω(SL) = 0.7 corresponds to the eutectic point of the EC–SL system [31].

**Figure 4:**
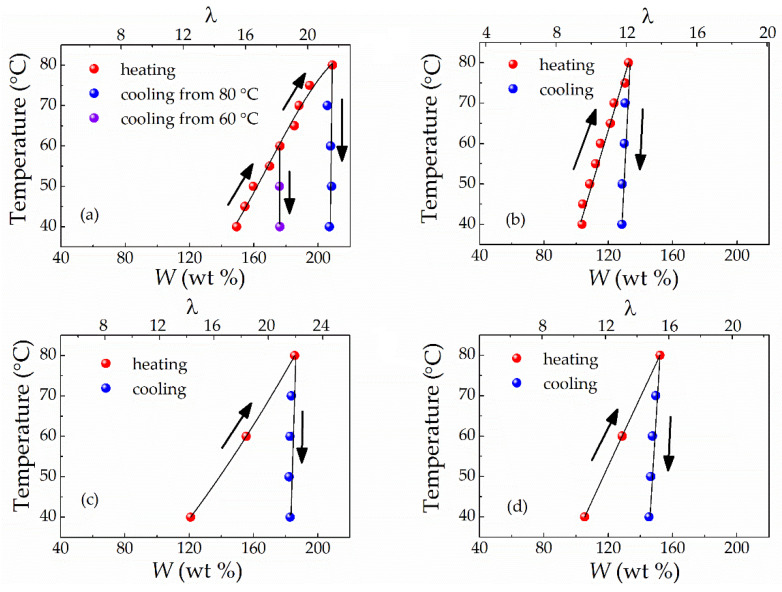
Dependence of the weight uptake (*W*) and molar uptake (λ) of a solvent of the Li-Nafion membrane vs. mixing temperature at heating and cooling mode for the following solvents: ethylene carbonate (EC) (**a**); sulfolane (SL) (**b**); EC/SL with ω(SL) = 0.2 (**c**); EC/SL with ω(SL) = 0.7 (**d**).

Measurements of the *W*–*T* dependencies were carried out by sequential heating (40 to 80 °C with 10 °C steps) and cooling (80 to 40 °C with 20 °C steps). Figure 4 displays the results obtained. It can be seen that the mixing temperature has a powerful effect on the solvent uptake of Li-Nafion regardless of the plasticiser composition: the higher the mixing temperature, the greater the solvent uptake. However, this characteristic was observed only in the heating cycle (Figure 4); during the cooling cycle, the decrease in solvent uptake was barely noticeable. As a result, the *W* value obtained for the highest temperature was virtually maintained for all other annealing temperatures at the cooling mode for all plasticiser compositions studied (Figure 4). Upon cooling from 80 °C, the *W* value for this temperature was almost preserved after thermal treatment at 60 and 40 °C. If cooling was carried out from 60 °C, again, a similar *W* value was retained after thermal treatment at 40 °C (Figure 4a). At the end of measurements, long-term (60 days) isothermal treatment was carried out at 40 °C to check that the equilibrium state of the swollen samples during the cooling cycle was achieved. However, this procedure did not influence the obtained W values. This observation suggests either that the samples are close to the equilibrium state or that syneresis requires a very long time.

The observed hysteresis in solvent uptake between the heating and cooling curves supports the hypothesis that an irreversible rearrangement of the supermolecular structure occurs under the influence of a plasticiser and elevated temperatures. Here, it should be noted that all membrane samples retained their integrity upon swelling despite significant mechanical properties changes.

The effect of temperature on equilibrium water content is well known for the protonated form of Nafion. Nevertheless, for H^+^ Nafion, the increase in *W* with temperature is much less pronounced (see, for example, data for Nafion 115 in Ref. [33]; for Nafion 212 and Nafion XL, Ref. [34]): within the same temperature range of 40 to 80 °C, the water uptake increased by less than 10% and *W* did not exceed 40%. Such a dramatic difference in behaviour upon swelling suggests significant variations in the molecular mechanisms of solvent absorption between the protonated Nafion–water and lithiated Nafion–EC/SL systems. To the best of our knowledge, the present work is the first to report the strong influence of temperature on the aprotic solvent uptake for lithiated Nafion and observe the heating-cooling hysteresis.

### 3.4. Thermal Analysis and Wide-Angle X-ray Diffraction of “Dry” Membranes

Despite having a large number of bulky side chains (Figure 1a), Nafion membranes are known to have significant levels of crystallinity [1,25,35]. This crystallinity is one of the most critical properties of Nafion, providing both its structural integrity and mechanical stability even at high levels of swelling [1]. As shown in Starkweather’s paper [35], the three-dimensional hexagonal structure of Nafion is similar to that of PTFE at elevated temperatures. Compared with PTFE, the X-ray diffraction peaks of Nafion are broadened and shifted toward smaller angles, indicating that crystals are smaller and less perfect [35,36]. It has been proposed that the lamellar morphology of crystallites is randomly distributed over the amorphous Nafion matrix [35,37,38]. For extruded Nafion membranes, the relative degree of crystallinity of about 7–17% is lower than that of PTFE (55–75%) [1]; the difference can be attributed to the effect of side chains and ionic groups hindering crystallisation [1,25,35].

**Figure 5:**
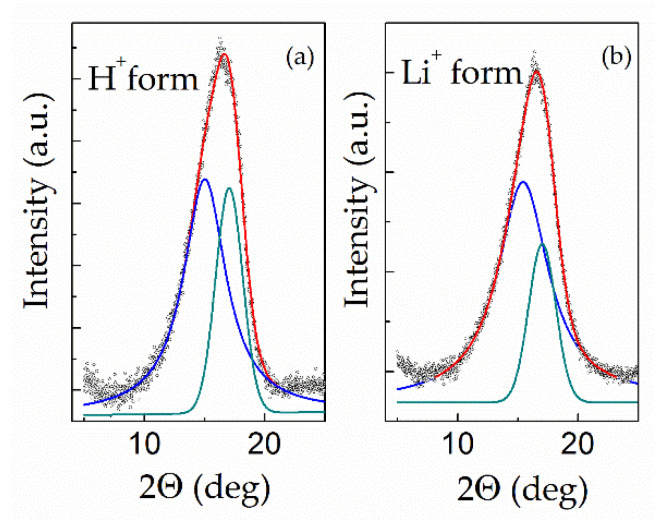
Wide-angle diffraction pattern of “dry” Nafion 115 membrane before (**a**) and after (**b**) lithiation.

In the present work, wide-angle X-ray diffraction was carried out as described in Section 2.5 for “dry” samples of Nafion 115 membrane in its H^+^ (as received) and Li^+^ forms in order to reveal the influence of the lithiation procedure (Section 2.1) on the degree of crystallinity and structural characteristics. Diffraction patterns of the samples in the 2θ range from 7 to 22° (where the most intensive peak is located) together with decomposed profiles are presented in Figure 5; their characteristics are shown in Table 1. For both samples, the measured curves were decomposed into a broad peak at 2θ ≈ 15° with a full width at half-maximum (fwhm) of ≈4° (amorphous halo), and a relatively sharp peak at 2θ ≈ 17° with fwhm of ≈2° (indexed as the (100) reflection of the hexagonal structure [35]). Taking into account the significant error in the decomposition of broadened profile, the WAXD results for H^+^ Nafion are in reasonable agreement with the literature data [1,25,35,36,37,38,39,40,41]. For lithiated Nafion, crystallographic data are extremely scarce (only Ref. [40] was found).

As shown in Table 1, the conversion of protonated Nafion into the lithiated form barely changed the most intensive crystalline reflection (100). However, the maximum of the amorphous halo shifted noticeably toward larger angles, while the degree of crystallinity (calculated by Equation (3)) significantly decreased as a result of lithiation.

The morphological features of the Nafion membrane, which contains crystalline inclusions and ionic clusters along with amorphous regions, clearly influence thermal transitions. It is known that crystalline regions of polymers at temperatures well below their melting point behave similarly to chemical crosslinking [42], while electrostatic interactions within ionic clusters act as physical crosslinking [1,43]. Limiting the segmental dynamics of Nafion macromolecules, all these factors are clearly manifested in its thermal behaviour. As mentioned in the review of Kusoglu [1], Nafion membranes exhibit three thermal transitions labelled α, β and γ, which correspond to distinct relaxation mechanisms. However, at present, even for the protonated form of Nafion, there is no consensus about their assignment. The γ relaxation around −120 to −90 (nearly independent of water absorption and cation type) is usually attributed to short-range molecular motions of the –CF_2_– backbone [1,40]; nevertheless, Corti et al. [44] reasonably reassigned this transition as the true glass transition temperature of the polymer. The β relaxation between −40 and +20 °C and the α relaxation between 90 and 120 °C (both sensitive to cation type and water content) were attributed to glass transition temperatures of the polar regions (ionic clusters) and non-polar matrix of the Nafion membrane. However, there is no consensus in the assignment of α and β relaxation to the former or latter process (see, for example, Refs. [1,41,42,43,44,45,46,47,48]). Moreover, the transition temperatures do not always fall within the indicated limits; they are also influenced by residual water (see, for example, work of Almeida and Kawano [43]). The melting point of H^+^ Nafion determined by DSC was about 275 °C [25]. It is significant that the melting in Nafion occurs over a very broad temperature range below 275 °C [25]. Therefore, thermal transitions in H^+^ Nafion above 200 °C are usually associated with crystalline melting [43,46,47,48]. The replacement of mobile protons by alkali metal cations increases both α and β relaxation temperatures by over 100 °C, while the γ relaxation temperature is around 100 °C [1]. It should be noted that for some ionic forms of Nafion, the β relaxation occurs as a shoulder on the low-temperature side of the α peak [44]. However, very little is known about thermal transitions in Li-Nafion; differential scanning calorimetry (DSC) measurements observed relaxations at 81 °C [49] and 212 ± 18 °C [1].

**Figure 6:**
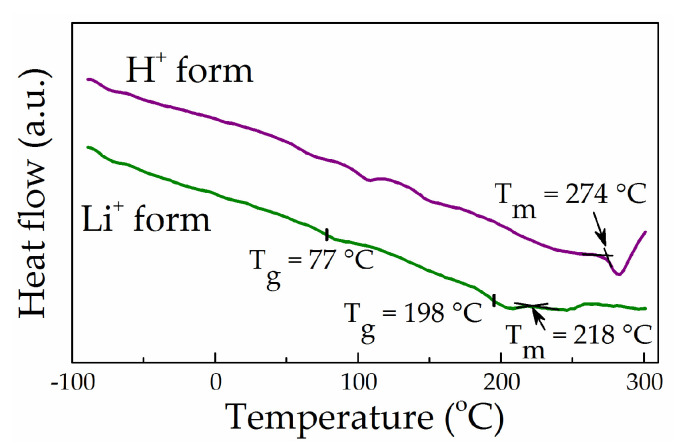
Differential scanning calorimetry (DSC) curves for the “dry” samples of Nafion 115 in H^+^ and Li^+^ forms.

In the present work, DSC measurements were carried out to analyse the thermal behaviour of lithiated Nafion compared with its protonated form. Figure 6 provides the DSC curves for the “dry” samples of Nafion 115 in H^+^ and Li^+^ forms. The region near –100 °C at the γ transition was not analysed due to instrumental effects. As can be seen from Figure 6, the DSC curve of H^+^ Nafion demonstrates thermal effects typical for a protonated membrane, including the well-defined endothermic effect of crystalline melting at 274 °C, which is entirely consistent with Gierke’s results [25] and our WAXD data (see Section 3.2).

For Li-Nafion, the DSC curve shows a transition near 77 °C, which is close to that reported by the group of Changchun Institute of Applied Chemistry [49], along with a diffused double effect at 198 and 218 °C (Figure 6). However, the available data did not allow us to identify the double peak at 198 and 218 °C reliably. The latter effect’s temperature is close to the value reported in the literature for the α relaxation in lithiated PFSA [1]. However, it seems more likely that the effect at 218 °C is a weak endothermic peak related to crystallite melting, while the effect at 198 °C may be attributed to α relaxation. Although a double effect also observed by Ozerin et al. [46] for the protonated form of PFSA at 227 and 239 °C was attributed by the authors to crystalline melting, the reasons for its splitting were not discussed in the cited work. DSC curves for Na, K, Rb, and Cs Nafion salts presented by Almeida and Kawano [43] exhibit a very similar diffused double effect, with the higher value attributed by the authors to crystallite melting. Considering the literature data, we tend to attribute the transition at 218 °C to crystal melting. A likely explanation for the remarkable decrease in the temperature from 274 °C (protonated form) to 218 °C (Li-Nafion) (Figure 6) is a reduction in the average size of crystallites resulting from the lithiation procedure.

### 3.5. Thermal Analysis and Wide-Angle X-ray Diffraction of Swollen Li-Nafion Samples

It is known that the morphology of an ionomer re-organises as the dry membrane absorbs a solvent; these structural changes following swelling can be observed by DSC and WAXD measurements. Therefore, the influence of a solvent on the thermal behaviour of lithiated Nafion was studied step-by-step during a gradual increase in solvent uptake. Ethylene carbonate was used as a solvent in these measurements. A weighted portion of EC was added to a piece of membrane to obtain a sample with a specified solvent uptake; after that, samples were stored for 24 h at the mixing temperature of 40 °C for homogenisation. An Li-Nafion sample saturated with EC at 40 °C was prepared as described in Section 2.3.

**Figure 7:**
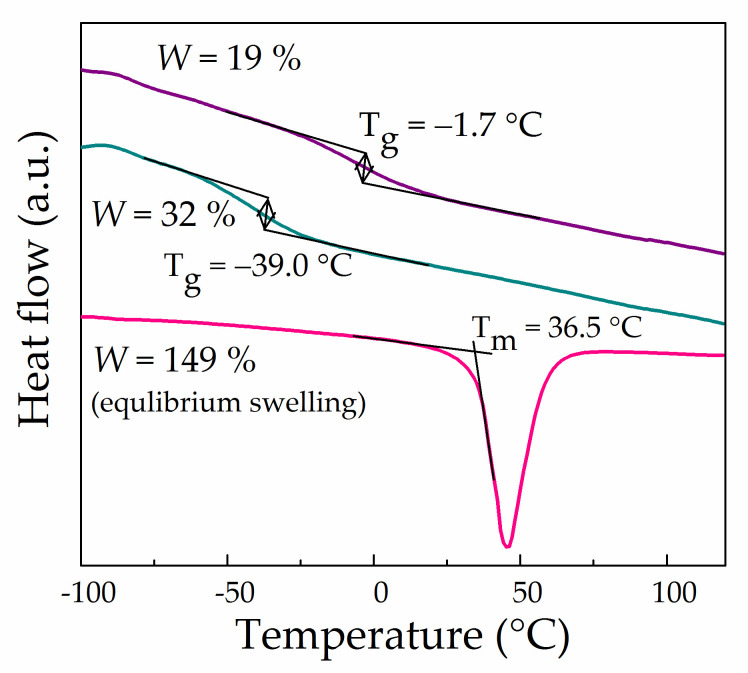
DSC curves for Li-Nafion with different contents of EC (40 °C/min).

Figure 7 demonstrates DSC curves for Li-Nafion samples having different EC contents. It can be seen that the introduction of a relatively small quantity of EC (*W* = 19%) led to an abrupt decrease in the glass transition temperature from 77 °C of a dry membrane to −2 °C without phase separation. With an increase in *W* to 32%, the glass transition temperature shifted even more to the negative region to reach −39 °C. Here, it is likely that saturation with EC (*W* = 132%) shifts the glass transition temperature of the membrane to below −80 °C (not observed in this work), while the DSC curve shows only the endothermic effect of the first-order phase transition at 36.5 °C associated with the melting of ethylene carbonate released from the membrane upon cooling to 25 °C (Figure 7).

The obtained results indicate that even pure EC, which is solid at room temperature, represents a very effective plasticiser for Li-Nafion. The destruction of ionic crosslinking due to the solvation of lithium cations by EC and charge screening releases segmental mobility of perfluorinated Nafion backbones. The structural rearrangements in the swollen membrane result from the decrease in *T*_g_ to very low values.

**Figure 8:**
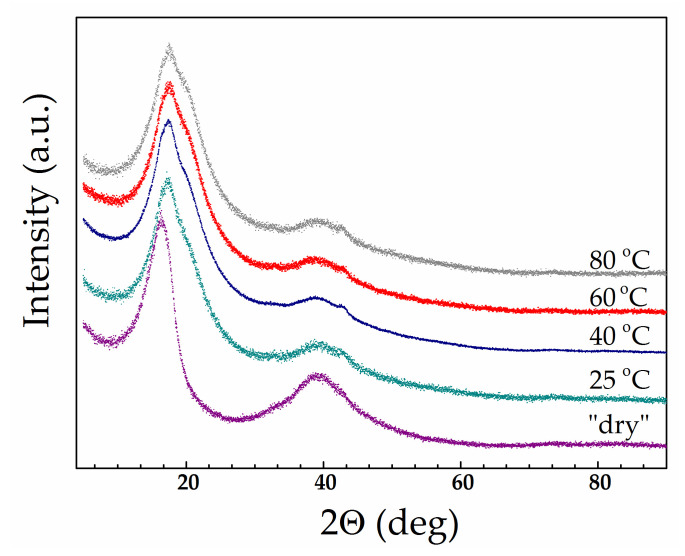
Wide-angle X-ray diffraction patterns for “dry” Li-Nafion and membrane samples swelled with EC/SL mixture with ω(SL) = 0.2 at different mixing temperatures specified in the figure.

The WAXD results fully support the assumption of structural changes. For the study, we selected samples saturated with a mixed solvent EC/SL with ω(SL) = 0.2 at various mixing temperatures: 25, 40, 60, and 80 °C. (Mixtures with ω(SL) = 0.2 were used for membrane swelling instead of pure EC due to being entirely homogeneous at 25 °C according to the EC/SL phase diagram [31]). Figure 8 shows diffractograms of swollen membranes having a diverse thermal history in comparison with a dry membrane. The appearance of a new shoulder on the side of large angles at the most intense peak (100) can be clearly seen.

**Figure 9:**
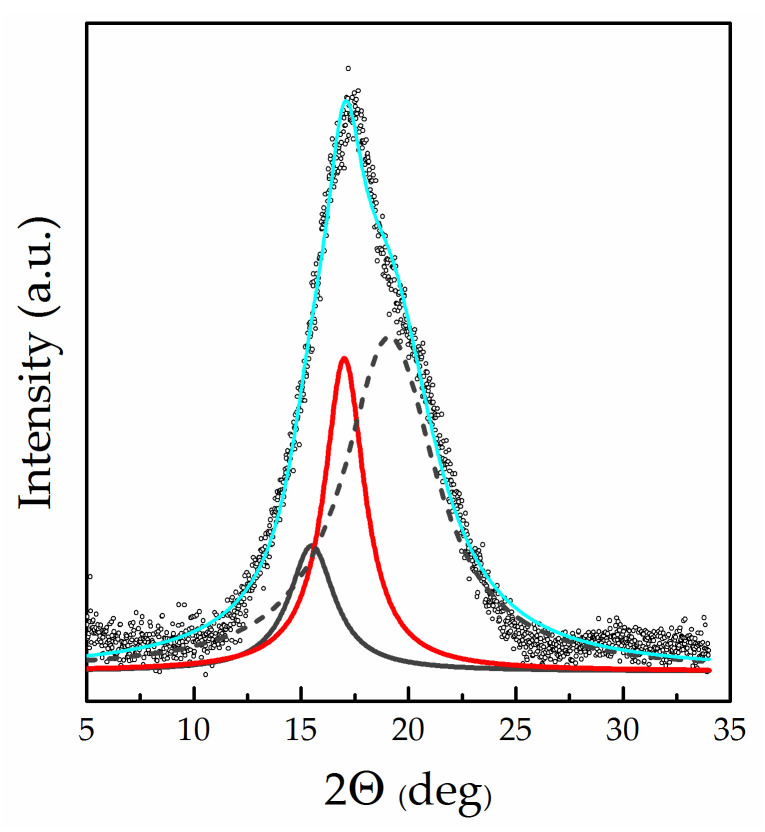
Decomposition of X-ray diffraction profile near (100) Bregg peak for Li-Nafion saturated with EC/SL mixture with ω(SL) = 0.2 at the mixing temperature of 25 °C.

**Figure 10:**
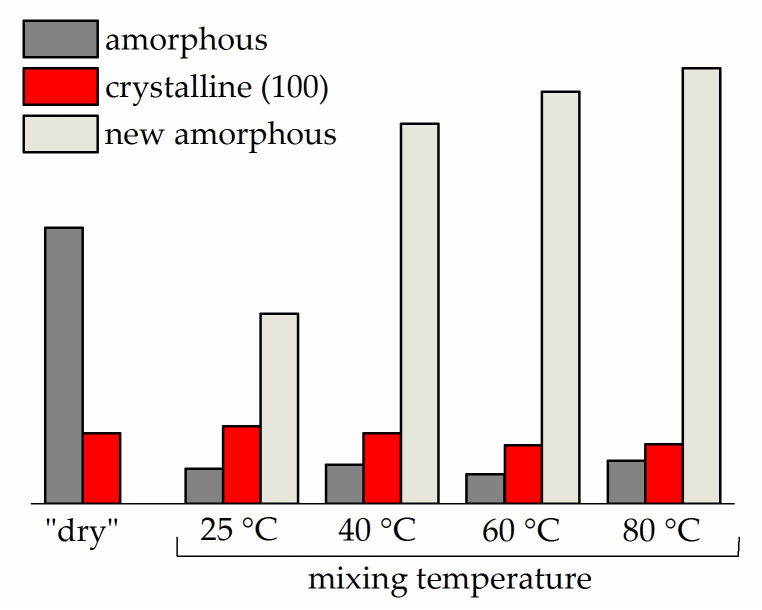
Amorphous and crystalline peak areas for Li-Nafion samples before and after swelling with an EC/SL mixture with ω(SL) = 0.2 at different mixing temperatures (specified in the diagram).

Figure 9 shows an example of the decomposition into components of the measured profile in the region 2θ = 7–33°. As can be seen from the figure, the saturation with the plasticiser causes the appearance of a new broad peak (new amorphous halo) having a maximum at 2θ = 19.1–19.4°, which was absent in the dry membrane WAXD profile (right shoulder in Figure 8). The intensity of this peak noticeably increases with mixing temperature (and, accordingly, with solvent uptake; see Figure 4). Simultaneously, the intensity of the amorphous halo, which is identical to that appearing in the diffraction pattern of a “dry” membrane (Figure 5), decreases significantly. The WAXD technique used in the present study (transmission mode, see Section 2.5) allows the changes in the intensity of all three components of the complicated diffraction peak in the (100) region to be directly compared. Figure 10 presents the component intensities as a function of the mixing temperature and data for the “dry” membrane for comparison. The diagram demonstrates that the saturation with the plasticiser somewhat reduces the intensity of the crystal reflection (100) compared to the solvent-free membrane; an increase in the mixing temperature in the range of 25–80 °C also leads to a slight decrease in its intensity. However, the influence of the plasticiser on the state of the amorphous regions is much more significant: the proportion of the halo observed in the “dry” membrane sharply decreases (Figure 10). A new, very intense halo with a maximum at 19.1–19.4°, which appeared due to the plasticiser sorption and noticeably increases with mixing temperature up to 60 °C does not change significantly at 80 °C.

Based on changes in *T*_g_ and diffraction profiles of swollen membranes, it can be assumed that the penetration of the solvent into the amorphous regions of the polymer results in a new amorphous structure that includes not only macromolecules but also low-molecular-weight solvent. Unlike water, molecules of some polar organic solvents can interact with PFSA chains and penetrate amorphous regions to weaken interchain interactions [4,45,50]. Moreover, it is known that saturation with water does not cause any noticeable changes in the Nafion diffraction profile [46].

The excellent preservation of a membrane’s crystalline regions upon saturation with a plasticiser regardless of mixing temperature is a highly significant factor. This fact is due to two reasons. Firstly, the high melting point of the crystallites (≈218 °C) is significantly above the studied temperature range (25–80 °C). Secondly, the higher chain packing density of the crystalline structure makes it much more resistant to dissolution [45]. Here, the integrity of the membrane is ensured by crosslinking due to crystalline regions even when very high degrees of swelling are achieved (>200 at 80 °C for EC); this also explains the apparent similarity of their characteristics with those of chemically crosslinked gels.

## 4. Conclusions

Mixed solvent EC/SL is a suitable plasticiser for Li-Nafion, since it provides high ionic conductivity (10^−4^ Sm cm^−1^ at room temperature) due to high solvent uptake. The degree of swelling of the membrane depends on the composition of the binary plasticiser and the mixing temperature; moreover, hysteresis occurs between the heating and cooling mode curves. The sorption of even small amounts of a plasticiser shifts the glass transition temperature of the ionomer from 77 °C to negative temperatures; at saturation with EC/SL, it decreases to below −80 °C. While the introduction of the plasticiser leads to the critical reorganisation of the amorphous structure of the ionomer, it does not significantly influence the crystalline regions, which remain nearly unchanged in the studied mixing temperature range of 25–80 °C. Due to their high melting point (about 218 °C), crystallites in Li-Nafion act as chemical crosslinking elements, maintaining the integrity of the membrane even at a very high solvent uptake (>200%) and making its characteristics similar to those of a chemically crosslinked gel. The obtained results provide a more in-depth insight into the physicochemical nature of the ionomer. Thus, Li-Nafion plasticised with EC/SL may be promising for use as a polymer electrolyte in developing a new generation of LIBs with enhanced safety.

## Figures and Tables

**Table 1:** X-ray characteristics of “dry” Nafion 115 membrane before and after lithiation.

Sample	Amorphous Galo		(100)		Crystalline Degree, %
	2θ, Deg	Fwhm	2θ, Deg	Fwhm	
Nafion 115 (H^+^)	15.00 ± 0.10	4.29	17.03 ± 0.04	2.25	29
Nafion 115 (Li^+^)	15.41 ± 0.06	5.14	17.01 ± 0.03	2.27	20

## Data Availability

Not applicable.
